# Cross-Cultural Adaptation and Psychometric Properties of the Spanish Version of the Tampa Scale for Kinesiophobia for Temporomandibular Disorders

**DOI:** 10.3390/jcm9092831

**Published:** 2020-09-01

**Authors:** Roy La Touche, Joaquín Pardo-Montero, Ferran Cuenca-Martínez, Corine M Visscher, Alba Paris-Alemany, Ibai López-de-Uralde-Villanueva

**Affiliations:** 1 Departamento de Fisioterapia, Centro Superior de Estudios Universitarios La Salle, Universidad Autónoma de Madrid, 28023 Madrid, Spain; joaquinp@lasallecampus.es (J.P.-M.); fecuen2@gmail.com (F.C.-M.); albaparis81@yahoo.es (A.P.-A.); 2 Motion in Brains Research Group, Institute of Neurosciences and Movement Sciences (INCIMOV), Centro Superior de Estudios Universitarios La Salle, Universidad Autónoma de Madrid, 28023 Madrid, Spain; 3 Instituto de Neurociencia y Dolor Craneofacial (INDCRAN), 28003 Madrid, Spain; 4 Department of Orofacial Pain and Dysfunction, Academic Centre for Dentistry Amsterdam, University of Amsterdam and VU University Amsterdam, 1081 Amsterdam, The Netherlands; visscher@acta.nl; 5 Department of Radiology, Rehabilitation and Physiotherapy, Faculty of Nursing, Physiotherapy and Podiatry, Complutense University of Madrid, 28040 Madrid, Spain; ibai.uralde@gmail.com

**Keywords:** fear of movement, temporomandibular disorders, kinesiophobia, psychometric properties

## Abstract

The aim was to perform a translation, cross-cultural adaptation, and psychometric evaluation of the Spanish version of the Tampa Scale of Kinesiophobia for Temporomandibular Disorders (TSK-TMD-S). The study sample included 110 patients with TMD. We translated and cross-culturally adapted the TSK-TMD-S using standard methodology and analysed its internal consistency, test-retest reliability, construct validity, floor and ceiling effects, and discriminant validity. Confirmatory factor analysis extracted two factors and 10 items deemed essential for the scale. The TSK-TMD-S demonstrated good internal consistency (Cronbach’s α of 0.843, 0.938, and 0.885 for the entire scale, activity avoidance subscale, and somatic focus subscale, respectively; intraclass correlation coefficient, 0.81–0.9). No floor or ceiling effects were identified for this final version of the scale. The TSK-TMD-S total score showed moderate positive correlation with the craniofacial pain and disability inventory, visual analogue scale, general TSK and pain catastrophizing scale, and a moderate negative correlation with maximal mouth-opening. The receiver operating characteristic curve analysis showed that the subclassification employed for the TSK-TMD-S discriminates different kinesiophobia levels with a diagnostic accuracy between sufficient and good. The optimal cut-off point for considering kinesiophobia is 23 points. TSK-TMD-S appears to be a valid and reliable instrument for measuring kinesiophobia in patients with TMD.

## 1. Introduction

The term “craniomandibular pain” is increasingly employed to refer to the various diseases that can provoke craniofacial pain such as diseases of the temporomandibular joint (TMJ), classically defined as temporomandibular disorders (TMD) [1].

Masticatory system disease is complex and involves the mandible and cranial structures from which articular, muscular, and neural pain originate and can radiate to local or distant structures [2]. A study by Slade et al. found that 73% of cases of TMD presented combined arthralgia and myalgia, while 23% presented myalgia in isolation [3]. Patients with TMD also report intra-articular disorders, presenting various TMJ sounds (clicking, popping, and crepitus) associated with any TMJ movement [4]. Another presentation of craniomandibular disorder includes symptoms such as headache, otalgia, and tinnitus [5].

Fillingim et al. investigated the impact of psychosocial aspects on TMD prognoses and treatment outcomes [6]. The importance of these aspects has been recognized by the Diagnostic Criteria for TMD [4,7]. Axis II of these criteria is aimed at evaluating the psychological and social aspects of pain, focussing on pain intensity, mood depression, anxiety, pain-related disability, and parafunctional behaviours.

The term “kinesiophobia” refers to an intense fear of performing certain actions that might lead to injury or re-injury, which is accompanied by avoidance of real or anticipated situations that objectively never or almost never justify the behavioural response [8]. The aversive stimulus (in this case, the specific motor gesture) is programmed to present at some point in the future, and the instrumental (behavioural) response prevents the presentation of the aversive stimulus. This avoidance response leads to negative reinforcement [9] because the aversive event is avoided by engaging in the behaviour. This avoidance behaviour can be active or passive [9].

The Tampa Scale of Kinesiophobia (TSK) is a questionnaire developed to measure fear of movement [10]. The fear avoidance beliefs questionnaire and the TSK are commonly used to assess fear avoidance beliefs and fear of pain and re-injury in various conditions [11,12]. These scales have been translated into numerous languages and validated for a variety of patient conditions. Their psychometric properties have been tested in various languages and for numerous diseases [13,14]. TSK measures the belief that painful activity will result in damage, which is consistent with the construct of kinesiophobia. In addition, Bunzli et al. [15] showed a second belief associated with TSK: the belief that “painful activity will increase suffering/and or functional loss”. Gil-Martínez et al. [16] and Heuts et al. [17] found that kinesiophobia is a predictor of disability and craniofacial pain in patients with TMD.

The TSK for TMD (TSK-TMD) was developed by Visscher et al. [18] to assess kinesiophobia in patients with TMD. The TSK-TMD uses a Likert scale (1–4) containing 12 items in 2 domains, with higher scores indicating higher levels of kinesiophobia. An international expert group recently qualified this instrument as one of the most useful tools for physiotherapists who specialise in TMD, [19] and there has been growing interest in applying the existing tools to various cultures and languages. At present this instrument has been translated cross-culturally into 4 languages. For example, He et al. [20], validated the Chinese version of the TSK-TMD, while Park et al. [21] validated the Korean version of the TSK-TMD, both versions showing adequate psychometric properties.

To date, however, a Spanish version of the TSK-TMD has not been available. Given the lack of a Spanish-validated version of this scale for patients with TMD pain, the aim of this study was to translate, cross-culturally adapt and psychometrically evaluate the Spanish version of the TSK-TMD (TSK-TMD-S).

## 2. Methods

This study employed an observational, cross-sectional, descriptive design for the psychometric validation and transcultural adaptation of the TSK-TMD-S. A consecutive convenience sample of 110 participants was recruited from outpatients of private clinics specializing in craniofacial pain and TMD in Madrid, Spain.

Patients were selected if they met all of the following criteria: (1) painful TMD, the diagnosis of which was based on the Diagnostic Criteria for TMD [4], (2) pain history of at least 3 months prior to the study, (3) at least 18 years of age, and (4) good understanding of the Spanish language. The exclusion criteria were: (1) cognitive impairment, and (2) presence of psychiatric limitations that impede participation in the study assessments.

The study was conducted in 2 phases: (1) the application of a structured transcultural adaptation protocol to determine the scale’s linguistic validity, and (2) the evaluation of the scale’s psychometric properties. All research procedures were established in accordance with the Declaration of Helsinki and were approved in advance by the Bioethics Committee of the La Salle University Study Centre (CSEULS-PI-005/2020). Authorization for translation and cross-cultural adaptation was requested from the author who created the original version of the TSK-TMD [18].

### 2.1. Tampa Scale for Kinesiophobia for Temporomandibular Disorders

The TSK-TMD is an 18-item self-report questionnaire that assesses kinesiophobia [18]. The answer to each question is scored on a 4-point Likert scale using the following responses: strongly disagree (1), somewhat disagree (2), somewhat agree (3), and strongly agree (4). The scores are summed to provide a total score, with higher values reflecting greater kinesiophobia. After a confirmatory analysis, Visscher et al. [18] found that a two-factor model based on 12 items had the best fit for the TSK-TMD, with activity avoidance and somatic focus as its subscales.

### 2.2. Cross-Cultural Translation and Adaptation

The first phase of this study was conducted according to the conventional recommendations described by Wild et al. [22] for translating and culturally adapting questionnaires based on patient-reported measures.

1. Two native bilingual speakers with basic knowledge of the terminology employed in health sciences independently translated the original English version of the TSK-TMD into Spanish.
2. An expert panel on craniofacial pain and TMD (fluent in English and Spanish) evaluated, approved and merged the two translations of the scale. Two bilingual native speakers, who were unaware of the original version, then back-translated the TSK-TMD into English.
3. The semantic equivalence and content validity of the retro-translated version was confirmed by a judging panel of 8 experts, including specialists in psychometry, psychology, and TMD. The expert judging panel were asked to qualitatively assess each item (degree of comprehension, adequacy of wording) and quantitatively assess the items according to the following characteristics: (1) clarity (the item is easily understood, and its semantics and syntax are adequate); (2) coherence (the item is related to the factor being measured); and (3) relevance (the item is essential and should be included).
4. Once the information extracted from the expert judging panel was established and analysed, a pilot test was conducted to evaluate the understanding and clarity of the instrument in 15 patients with TMD.
5. Results of the pilot test were employed to make final changes to the instrument, thus concluding the linguistic adaptation process with the results of the final version to be employed in the second phase of evaluating the psychometric properties.

### 2.3. Data Analysis

The procedures to determine the theoretical construct and evaluate its reliability and external validity are detailed below. Prior to the validity assessment, we conducted data screening and item analysis to examine the items’ suitability. According to Tabachnick and Fidell [23], non-discriminatory items (80% or more of the respondents responded identically) and items with greater than 50% of missing values were inspected and removed if they were considered not critical for the analysis. We employed SPSS software version 21 (IBM SPSS Statistics) and Mplus statistical software 7.11 for all statistical analyses and set the statistical significance at 5% (*p* < 0.05).

#### 2.3.1. Content Validity

We calculated the content validity of the pre-final version of the TSK/TMD-S using the Aiken *V* test [24] whose values range from 0 to 1; values < 0.70 are considered rejected and are not ultimately included [25].

#### 2.3.2. Construct Validity

We evaluated the construct validity using a 2-step process: (1) exploratory factor analysis (EFA) to determine the optimal factor structure, and (2) confirmatory factor analysis (CFA) to confirm the theoretical factor structure of the proposed model. The sample size needed to ensure the proper functioning of the statistical analysis of the construct validity is controversial. We therefore established sample-size-based criteria from several authors, who proposed a minimum of 50 observations and a recommended sample size of at least 100 [26,27,28,29]. In our study, we opted for the latter option, with a total sample of 120 participants.

To determine whether the Pearson’s correlation matrix was factorizable, we assessed Bartlett’s test and the Kaiser–Meyer–Olkin (KMO) test [23]. We established the optimal number of factors based on Kaiser’s eigenvalue criterion (eigenvalue ≥ 1) and evaluation of the scree plot [30]. These criteria were confirmed using the parallel analysis method, according to statistical recommendations [31,32]. In EFA, there are differing opinions regarding the optimal method for factor analysis extraction [33]. To avoid a priori assumptions, we employed 3 different factor extraction methods (maximum likelihood, principal axis factoring, and principal component analysis) with both varimax and oblique rotation. A factor loading greater than 0.4 was considered necessary for the item’s inclusion in each factor.

We performed the CFA to validate our exploratory and preliminary solution using a more rigorous model than that used for the EFA because we adjusted a congeneric factor solution. Specifically, we used the weighted least squares mean, the variance adjusted estimation method, and the following goodness-of-fit indices: (1) comparative fit index (CFI) and Tucker Lewis index (TLI) as comparative fit indices, (2) the root mean square error of approximation (RMSEA) as a parsimony fit index, and (3) the chi-squared and weighted root mean square residual (WRMR) as absolute fit indices. To determine an acceptable model fit, we employed Hu and Bentler criteria (TLI ≥ 0.95, CFI ≥ 0.95, RMSEA ≤ 0.06, and WRMR ≤ 1.0) [34]. We calculated modification indices to detect local mis-specified areas of the model not sensitive to the previously mentioned overall goodness-of-fit indices [35].

#### 2.3.3. Reliability

We assessed the reliability, internal consistency, and test-retest reliability (responsiveness) of the TSK-TMD-S. Specifically, we employed the Cronbach’s α > 0.70 criterion [36] for internal consistency. We examined test–retest reliability using the intraclass correlation coefficient (ICC) and considered a value greater than 0.70 acceptable [37]. We measured the precision of the test–retest reliability of the TSK-TMD-S with the standard error of measurement (SEM), which was calculated as follows: $SD\times \sqrt{1-ICC}$ (where *SD* is the standard deviation) [38].

In addition, the minimum detectable change (MDC) was calculated to establish if the magnitude of change observed between the 2 measures (separated by 7–10 days) reflects real change and not just measurement error [39]. The MDC at the 90% confidence interval (MDC_90_) and MDC_95_ were calculated as $SEM\times \sqrt{2}\times 1.65$ and $SEM\times \sqrt{2}\times 1.96$, respectively.

#### 2.3.4. Floor and Ceiling Effects

The floor/ceiling effect was evaluated by calculating the percentage of patients obtaining the minimum or maximum possible scores on the TSK-TMD-S. If at least 15% of the patients achieved the minimum/maximum score, the floor/ceiling effect was considered to be present [37].

#### 2.3.5. Convergent Validity

We analysed the convergent validity using Pearson correlations between TSK-TMD-S and the other pain-related measures: the Craniofacial Pain and Disability Inventory (CF-PDI), the Spanish version of the TSK, the Spanish version of the Pain Catastrophizing Scale (PCS), the visual analogue scale (VAS), and the maximal mouth opening (MMO). A value <0.30 was considered a low correlation, 0.30–0.60 a moderate correlation, and >0.60 a strong correlation [37].

- CF-PDI was employed to measure pain, disability, and the functional status of the jaw and craniofacial regions. The CF-PDI consists of 21 items with 4 possible answers for each item and has good structure, internal consistency, reproducibility, and construct validity and is therefore an objective tool for assessing pain and disability in patients with craniofacial pain [40].
- We assessed pain catastrophizing using the Spanish version of the PCS [41], which has shown good psychometric properties [41] and consists of 13 items divided into 3 domains: rumination, magnification, and helplessness.
- We assessed fear of movement using the Spanish version of the TSK [11]. Total TSK scores range from 11 to 44 points, and higher scores indicate greater fear of pain, movement, and injury. The TSK has a two-factor structure of activity avoidance and harm and has been demonstrated to have acceptable psychometric properties [11].
- We measured pain intensity with the VAS, which consists of a 100 mm horizontal line with pain descriptors indicating “no pain” on the left side and “the worst pain imaginable” on the right side. The VAS is a reliable and valid measurement of pain [42].
- We assessed MMO with the craniomandibular scale. The distance between the superior incisor and the opposite inferior incisor was measured in mm with a craniomandibular scale (Pat. No. ES 1,075,174 U, INDCRAN: 2011. INDCRAN, Madrid, Spain). This procedure has high inter-rater reliability [43].

#### 2.3.6. Discriminant Validity

We employed the discriminant validity of the TSK-TMD-S to assess the various levels of jaw kinesiophobia as a criterion variable for item 14 (“Do you fear moving your jaw?”) of the CF-PDI. We scored this item with the following Likert scale: (1) no fear of moving the jaw (subclinical), (2) mild fear of moving the jaw, (3) moderate fear of moving the jaw, and (4) severe fear of moving the jaw.

We applied a one-factor ANOVA with Bonferroni correction to determine the differences between fear levels with respect to the TSK-TMD-S results and calculated the percentage for each level. We evaluated the area under the receiver operating characteristics curve to determine the proportion of patients correctly classified at the various levels. The highest value for this parameter is 1. Thus, the closer the result is to this value, the better the diagnostic utility. Diagnostic accuracy is considered excellent for a result of 0.9–1, very good for 0.8–0.9, good for 0.7–0.8, sufficient for 0.6–0.7, and poor for 0.5–0.6. If the result is <0.5, the test is considered not useful [44].

We calculated the optimal cut-off point between levels of jaw kinesiophobia using the Youden index [45], as well as the following indicators in diagnostic tests for each score: sensitivity, specificity, negative predictive value, and positive predictive value.

## 3. Results

The translation-retranslation phase showed a good equivalence with the original scale, and we made very few semantic changes. In terms of content validity, the quantitative analysis showed that 7 items obtained values <0.70; we therefore decided to remove them from the pilot test version.

When assessing the participants’ understanding during the pilot test, we detected no limitation in answering the items of the TSK-TMD-S. The mean time for completing the TSK/TMD-S was 136 ± 40 s, with a response rate of 100%.

The total sample was composed of 110 participants, 67.3% of whom were women (74 women and 36 men). Most patients were diagnosed with pain attributed to myofascial pain alone or combined with arthralgia (mixed diagnosis) (28.2% in both cases). The remaining patients had temporomandibular joint arthralgia (19.1%) or headache attributed to TMD (24.5%). Table 1 presents the patients’ sociodemographic characteristics, maximal mouth opening, and scores on the various self-reported scales.

### 3.1. Exploratory Factor Analysis

Prior to the exploratory factor analysis, we calculated Cronbach’s α coefficient for the entire scale (α of 0.825) and the adjusted item-total correlations (mean item-total correlation of 0.482)**.** We removed item 11 for several reasons: (1) the item had a low item-total correlation (<0.400), (2) the α coefficient increased by removing it from the scale (α of 0.843), and (3) when we performed the preliminary exploratory factor analysis with the 11-item version, its factor loading was <0.300. 

The KMO test showed acceptable data suite for factor analysis (KMO score of 0.754), and Bartlett’s test of sphericity rejected the identity matrix null hypothesis (χ^2^ (45) = 956.54, *p* < *0*.001).

Based on these results, continuing with the EFA would be justified. Lastly, we used the principal axis method of factor extraction with oblimin rotation, because we considered it the most appropriate and the results with the remaining methods (maximum likelihood, principal component analysis, and orthogonal rotation) were very similar. The scree-plot, Kaiser’s criteria, and the parallel analysis with the polychoric correlation matrix suggested retaining 2 factors. Thus, all the criteria converged on the 2-factor solution, which together represented 75.2% of the total variance. The first factor (43.36% of the total variance) consisted of items 5 through 9. The theoretical contents of this factor refer to the patients’ belief that jaw activity/movement will cause (re)injury or increased pain (factor 1 was called “activity avoidance”). The second factor (31.81% of the total variance) consisted of items 1, 2, 3, 4, and 10 and was named “somatic focus” because it mainly focused on the patients’ beliefs/emotions regarding their serious underlying medical issues. The factor loading of each item is shown in Table 2.

### 3.2. Confirmatory Factor Analysis 

**Figure 1:**
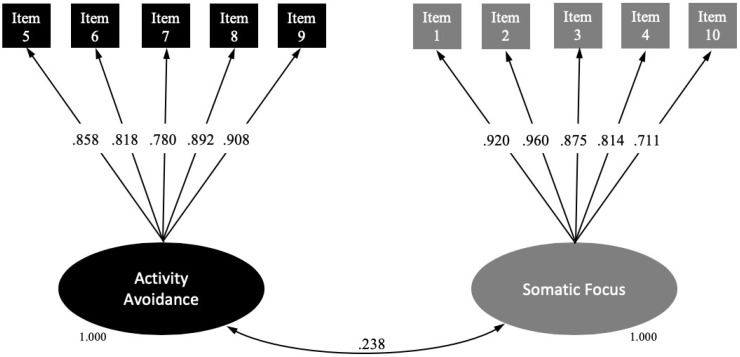
Structural equation modelling for the final Spanish version of the TSK-TMD.

All items were encompassed within the theoretical factor that was assumed and had an optimal factor loading (>0.700) in their corresponding factors. We therefore decided to fit a 2-factor CFA model with the aforementioned 10 items. The model fitted the data reasonably well (χ^2^ (34) = 56.59; *p* = 0.0088; CFI, 0.989; TLI, 0.986; RMSEA, 0.078; 95% CI 0.039–0.112; WRMR, 0.681). Figure 1 shows the standardized factor loadings of the final 2-factor solution. The observed indicators were reliable measures of their scales, given that all of the standardized factor loadings were >0.700. Factors 1 (activity avoidance) and 2 (somatic focus) were slightly correlated (*r* = 0.238; *p* = 0.012), indicating that both factors evaluate the same theoretical construct from different perspectives.

### 3.3. Reliability

The internal consistency of the total scale was 0.843 (95% CI 0.795–0.883), with its two subscales showing an internal consistency >0.800 (Activity avoidance: 0.938; 95% CI 0.918–0.955. Somatic focus: 0.885; 95% CI 0.847–0.916). To assess the test–retest reliability of the instrument, 85 patients (66.7% women; age, 46.04 ± 13.31 years; body mass index, 25.45 ± 4.14 kg/m^2^; duration of the disorders, 74.25 ± 55.54 months) re-took the scale 10 days later. According to the ICC, the scale’s stability over time was excellent, with an MDC_95_ of 6.25. Table 3 shows the descriptive statistics and results of the test–retest reliability and responsiveness analysis for the TSK-TMD-S and its subscales.

### 3.4. Final Version: Floor and Ceiling Effects

The final version of the TSK-TMD-S scale consisted of 10 items formulated directly/positively and distributed across the 2 subscales as follows: (1) somatic focus (items 1, 2, 3, 4, and 10) and (2) activity avoidance (items 5 through 9). The total score on the final version of the TSK-TMD-S can range from 10 to 40 (somatic focus, 5–20; activity avoidance, 5–20), with higher scores indicating greater fear of jaw movement and re-injury. We identified no floor or ceiling effects in this final version of the scale given that only 3.6% of the patients achieved the lowest possible score, and none had the highest possible score (somatic focus: floor effect of 11.8%, ceiling effect of 0.9%; activity avoidance: floor effect of 12.7%, ceiling effect of 2.7%).

### 3.5. Convergent and Discriminant Validity

The total score of the TSK-TMD-S showed a positive, significant, and moderate correlation with the CF-PDI, VAS, the general TSK, and PCS. The TSK-TMD-S was also significantly, negatively, and moderately correlated with MMO. In general terms, the activity avoidance subscale presented a lower magnitude relationship with all the variables evaluated when compared with the somatic focus subscale. Table 4 shows the correlations between the TSK-TMD-S and its subscales on the one hand and all of the assessed self-reported scales and maximal mouth opening on the other.

**Figure A1:**
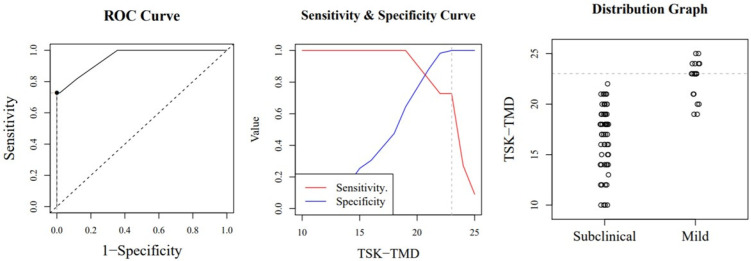
Optimal cut-off point between levels of jaw kinesiophobia. The image shows a ROC (Receiver Operating Characteristic) curve that represents the sensitivity of a diagnostic test that produces continuous results, depending on false positives (complementary to specificity), for different cut-off points (image on the **left**), the image where the cut-off point at which the highest sensitivity and specificity is achieved (image in the **middle**) and finally, a subclinical and mild sample distribution graph (image on the **right**).

**Figure A2:**
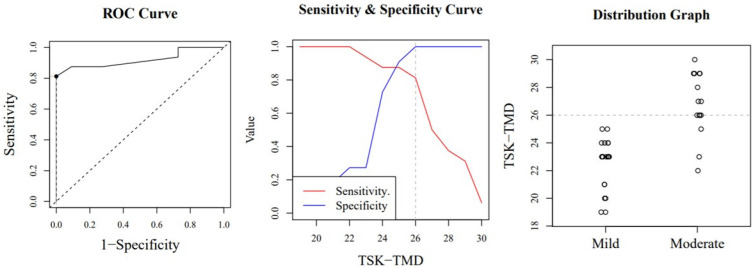
Optimal cut-off point between levels of jaw kinesiophobia. The image shows a ROC (Receiver Operating Characteristic) curve that represents the sensitivity of a diagnostic test that produces continuous results, depending on false positives (complementary to specificity), for different cut-off points (image on the **left**), the image where the cut-off point at which the highest sensitivity and specificity is achieved (image in the **middle**) and finally, a mild and moderate sample distribution graph (image on the **right**).

**Figure A3:**
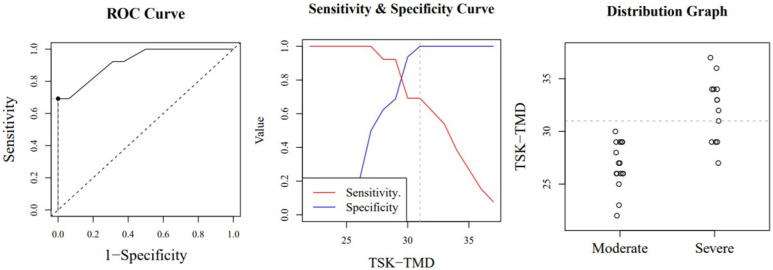
Optimal cut-off point between levels of jaw kinesiophobia. The image shows a ROC (Receiver Operating Characteristic) curve that represents the sensitivity of a diagnostic test that produces continuous results, depending on false positives (complementary to specificity), for different cut-off points (image on the **left**), the image where the cut-off point at which the highest sensitivity and specificity is achieved (image in the **middle**) and finally, a moderate and severe sample distribution graph (image on the **right**).

The ANOVA results show that there are differences in the various kinesiophobia levels (F = 132.35; *p* < 0.001). In the post hoc tests, we observed significant differences (*p* < 0.001) between each of the established levels. The highest percentage of patients was located in the subclinical level of kinesiophobia (53.64%). Table 5 shows the other percentages and descriptive statistics according to level. The TSK-TMD-S had excellent diagnostic accuracy (in terms of specificity) for classifying patients into 3 levels; however, the sensitivity was very good and good for mild and moderate cases, respectively, and sufficient for severe cases. The optimal cut-off point for considering kinesiophobia is 23 points. Table 5, Appendix A, Appendix B and Appendix C (Figure A1, Figure A2 and Figure A3) show the diagnostic accuracy results and all optimal cut-off points.

## 4. Discussion

This study’s main objective was to cross-culturally adapt the TSK-TMD-S into European Spanish. Our findings suggest that the 10-item, 2-factor Spanish version of TSK-TMD-S has adequate internal reliability, test-retest reliability, content validity, construct validity, and convergent validity.

The TSK-TMD-S covers all topics related to construct kinesiophobia in TMD and was analysed by experts to retain the items with the best properties. We added a psychometric content validity test (Aiken’s V) to quantify the instrument’s analysis. Experts were asked to evaluate each item’s relevance, clarity, and representativeness. Based on their assessments, we obtained a pool of 11 items from the original 18. The study sample consisted of 110 adult patients, with more women (67.3%) than men, as is reflected in the prevalence of TMD, the data of the original TSK-TMD, and the versions we examined (except for the Brazilian version, which was only tested on women).

The results of this study are quite similar to those of the original (Dutch) version of the TSK-TMD [18], the Brazilian version [46], the Chinese version [20], the Korean version [21], and the Japanese version [47].

### 4.1. Factorial Structure

Before conducting an exploratory factorial analysis (EFA), we removed item 11 (“I am afraid to open my mouth wide because then I might not be able to close it again”) for several reasons. Visscher et al. [18] considered the item essential for those patients with TMD who experience an open lock. In our sample, however, 59.1% answered “never” with respect to fear of jaw movement.

We performed an EFA to prevent biases before conducting a CFA. We did not proceed directly to the CFA to increase support for the findings of the original TSK-TMD. However, we expected to find a two-factor structure such as the one in the original TSK-TMD.

The exploratory EFA employed the parallel analysis technique, which is a demanding technique that produces more outcomes than are trustworthy. Furthermore, the principal component method is not a factor analysis method but rather a method to reduce the dimensions that reject measurement errors, an issue that is particularly important when the analysis is performed on the item scores. This practice frequently leads to overestimating factor loadings and the variance explained by the factors [48].

Regarding the internal structure of the TSK-TMD-S scores, the results of the exploratory factor analysis support a two-dimensional solution, with 2 factors explaining more than 75.2% of the total variance (as in the Chinese version) [20], which is more than is usually observed in EFA data in psychological questionnaires. When examining the original TSK-TMD-S, for example, the solution not only accounted for 48.4% of the explanatory variance but also supported the concept that the TSK-TMD comprises two distinct and separate aspects of kinesiophobia [18].

Activity avoidance (our most important factor) also appears in the Spanish version of the TSK (general TSK in the musculoskeletal sample) and is more important in terms of the explained variance than the second factor, with activity avoidance explaining 43.36% of the total variance with only 5 items. The theoretical content of this factor refers to the belief that jaw activity might cause a (re)injury or increased pain. Item 9 (similar to TSK 17) and item 5 (analogous to TSK item 10) are the most relevant for understanding this factor.

The second factor (somatic focus) accounted for 31.81% of the total variance and also had only 5 items. We kept the name “somatic focus”, although we doubt that the concept of “harm” in the general TSK fits the content better. This factor actually refers to the somatic focus on a potentially harmful event. It should be noted that item 1 “I am afraid that I might injure myself if I move my jaw” (similar to TSK item 1) has the greatest weight in the somatic focus scale; however, a similar TSK item appears in activity avoidance. 

In terms of TMD, the questionnaire’s factorial structure appears to be clearer than that of the general instrument created with a sample of musculoskeletal disorders. Each item fits its expected factor with no transformation or change, and its interpretation is therefore likely to be easier.

The subsequent CFAs provided strong evidence of two factors (RMSEA, 0.078; CFI, 0.989), and the correlation between the activity avoidance and somatic focus factors was only moderate (*r* = 0.24). The results for the TSK-TMD-S coincide with those of the other versions (Dutch, Chinese, Brazilian, and Japanese) [20,21,46,47]. Both factors evaluate the same theoretical construct from different perspectives. As with the original TSK, the Spanish version presents the robustness of a two-factor structure, which was also found with other patient groups, including patients with low back pain, fibromyalgia, and chronic fatigue syndrome [49,50,51]. The TSK-TMD-S showed good internal consistency (α of 0.84; 0.80 for activity avoidance and 0.89 for somatic focus), similar values to those reported for the original scale, with even higher values for somatic focus (0.66 for the original somatic focus). The test–retest reliability of the full TSK-TMD-S (ICC, 0.90) and the domain scores showed excellent values (ICC > 0.75), higher than those of the original TSK-TMD version 0.73 [18], the Chinese version 0.80 [20], and the Japanese version 0.89 [47]. The TSK-TMD-S has no reverse-scored items that represent a validity risk; the 4 inverse-scored items were not included in further analyses. Given that we identified no floor or ceiling effects for this final version of the scale and that both the SEM and MDC_95_ of the TSK-TMD-S were low, it might be possible that the TSK-TMD-S could be employed to distinguish different patient groups by their scores.

### 4.2. Convergent and Discriminant Validity

As expected, we found a significant correlation between TSK-TMD-S and TSK (0.56). TSK-TMD-S therefore measures kinesiophobia, although kinesiophobia is not manifested identically if different movements or anatomical regions are involved. In addition, as expected, the TSK-TMD-S correlated with all other measures, including the physical measure MMO.

Considering the subscales, however, somatic focus is closely related to the general TSK (0.51) and to a lesser degree with activity avoidance (0.28). Therefore, the type of activity avoidance measured by the TSK-TMD appears to be significantly different from that measured by the original TSK.

It is noteworthy that somatic focus shows a relationship with pain-related disability, CF-PDI. Strong evidence suggests that kinesiophobia is a predictor of disability in patients with various types of chronic pain, including TMD [17,52,53].

An interesting finding is that activity avoidance is not related to pain. Other psychological factors are probably responsible for maintaining this avoidance.

Numerous authors have reported that catastrophizing is more related to somatic focus than to activity avoidance [12,54]. which suggests a discriminative validity. Catastrophism is related to kinesiophobia but is a separate and logically related construct. Somatic focus represents the patient’s belief that the complaints are related to a serious medical problem, which is strongly associated with catastrophizing thoughts.

### 4.3. Reliability

According to the ICC, the scale’s stability over time was excellent (MDC_95_ of 6.25). Our study has a number of important strengths, as well as certain limitations. We did not assess the correlations between the TSK-TMD-S and depression. Symptoms of depression often occur in this population (see the Brazilian version of the TSK-TMD). Future longitudinal studies are warranted to establish the predictive power of kinesiophobia measured with the TSK-TMD-S to detect cases at risk of abandonment. The receiver operating characteristics curve analysis showed that the subclassification employed for the TSK-TMD-S discriminates different kinesiophobia levels with a diagnostic accuracy between sufficient and good. These data provide new diagnostic information that can help clinicians make better treatment choices. Of all the versions of the TSK-TMD scale published so far, the Brazilian Portuguese version identified a subclassification with its respective cut-off points. Unlike our study, the Brazilian study only employed 3 subdivisions and used a different statistical method to identify their values. Despite the differences, the cut-off points for the Brazilian and Spanish scales are very similar, especially considering that the Spanish version has 10 items and the Brazilian version has 12 items [55]. For the high classification level, the Brazilian scale found a cut-off point of 33 items, whereas we found 31 items. For the moderate classification, both the Brazilian scale and ours determined a score of 26.

## 5. Conclusions

The TSK-TMD-S appears to be a valid and reliable instrument for measuring kinesiophobia in patients with TMD.

## Figures and Tables

**Table 1:** Sociodemographic data and scores obtained on the self-reported scales.

Sociodemographic Data	Mean ± SD	Range (Min–Max)
Age (years)	45.58 ± 12.92	19–68
Height (cm)	162.75 ± 8.37	148–181
Weight (Kg)	66.94 ± 10.85	50–97
Visual Analogue Scale (100-mm)	52.73 ± 14.44	19–79
Maximal Mouth opening (mm)	41.7 ± 4.92	30–58
Craniofacial Pain and Disability Index		
Pain and Disability	15.54 ± 6.16	4–29
Jaw Functional Status	5.43 ± 3.52	0–15
Total Scale Score	20.96 ± 8.72	4–40
Tampa Scale of Kinesiophobia-11		
Harm	9.1 ± 2.62	4–15
Activity Avoidance	15.73 ± 4.69	7–25
Total Scale Score	24.83 ± 6.79	11–37
Pain Catastrophizing Scale		
Rumiation	7.93 ± 3.14	1–15
Magnification	5.47 ± 2.31	1–11
Helplessness	9.56 ± 4.1	2–20
Total Scale Score	22.96 ± 8.16	7–40
TSK-TMD (Final version, 10-items)		
Somatic Focus	11.06 ± 4.3	5–20
Activity Avoidance	10.46 ± 3.79	5–20
Total Scale Score	21.26 ± 6.09	10–37
**Categorical Variables**	***n* (%)**	
Employment status		
Employed	49 (44.5)	
Unemployed	26 (23.6)	
Student	16 (14.5)	
Retired	3 (2.7)	
Medical leave due to disability	16 (14.5)	
Level of education		
Primary education	37 (33.6)	
Secondary education	48 (43.6)	
University education	25 (22.7)	

Abbreviations: TSK-TMD, Tampa Scale of Kinesiophobia-Temporomandibular Disorders.

**Table 2:** Exploratory factor analysis solution.

Item	Factor 1 (Activity Avoidance)	Factor 2 (Somatic Focus)
1. Tengo miedo de que si muevo la mandíbula pueda hacerme daño. I am afraid that I might injure myself if I move my jaw.	0.168	0.914 *
2. Si ignoro el dolor de la mandíbula, éste se verá incrementado. If I ignore my jaw symptoms, they would get worse.	0.175	0.750 *
3. Mi mandíbula me está indicando que hay algo mal en ella. My jaw is telling me that something is seriously wrong with it.	0.108	0.754 *
4. Mis síntomas mandibulares indican que hay una lesión. My jaw symptoms mean that I have injured my jaw.	0.124	0.772 *
5. La manera más segura de evitar que mis síntomas empeoren es tener cuidado y no mover la mandíbula más de lo necesario. The safest way to prevent my symptoms from getting worse is to be careful and not to move my jaw any more than necessary.	0.936 *	0.062
6. Mis síntomas mandibulares me hacen saber cuándo debo dejar de mover la mandíbula para que no empeore o me haga daño. My Jaw symptoms let me know when to stop moving my jaw so that I do not injure.	0.815 *	0.105
7. No es seguro mover la mandíbula cuando se tiene alguna afectación en ella. No one should have to move the jaw when he/she has a jaw problem.	0.826 *	0.216
8. No debo realizar las funciones que normalmente hace una persona con su mandíbula, porque podría lesionarme la mandíbula e incrementar los síntomas. I cannot do everything other people can do, because it is too easy for me to injure my jaw.	0.812 *	0.172
9. Nadie debe mover la mandíbula si tiene problemas en la mandíbula. It is really not safe for someone with a jaw condition like mine to use the mouth a lot.	0.964 *	0.064
10. No debería dolerme si no hubiese nada dañado en la mandíbula. I would not have this many jaw symptoms if there was not something potentially harmful going on.	0.003	0.723 *
11. Tengo miedo de abrir la boca, porque es posible que no pueda volver a cerrarla. I am afraid to open my mouth wide because then I may not be able to close it again.	-	-

Note: * = *p* < 0.05.

**Table 3:** Descriptive statistics, intraclass correlation coefficients (ICCs) and associated 95% confidence intervals (CIs), standard error of measurement (SEM), minimal detectable change (MDC_90_) and MDC_95_.

	Mean ± SD		ICC (95% CI)	SEM	MDC_90_	MDC_95_
	Test 1	Test 2				
TSK-TMD-S	21.60 ± 5.77	21.24 ± 5.00	0.904 (0.853 to 0.938)	2.25	5.26	6.25
Somatic Focus	11.37 ± 4.32	10.86 ± 3.68	0.854 (0.776 to 0.905)	2.01	4.69	5.57
Activity Avoidance	10.58 ± 3.49	10.38 ± 2.96	0.809 (0.707 to 0.876)	1.84	4.28	5.09

TSK-TMD-S, Tampa Scale of Kinesiophobia-Temporomandibular Disorders.

**Table 4:** Convergent validity of the TSK-TMD-S.

	TSK-TMD		
Convergent Validity	Total Score	Somatic Focus	Activity Avoidance
CF-PDI Pain and Disability Jaw Functional Status Total Scale Score	0.421 ** 0.530 ** 0.511 **	0.594 ** 0.471 ** 0.610 **	0.104 0.378 ** 0.226 *
TSK-11 Harm Activity Avoidance Total Scale Score	0.587 ** 0.487 ** 0.563 **	0.624 ** 0.509 ** 0.593 **	0.269 ** 0.251 ** 0.277 **
Pain Catastrophizing Scale Rumination Magnification Helplessness Total Scale Score	0.315 ** 0.428 ** 0.340 ** 0.413 **	0.438 ** 0.454 ** 0.447 ** 0.521 **	0.057 0.290 ** 0.160 0.197 *
Visual Analogue Scale	0.335 **	0.425 **	0.088
Maximal Mouth Opening	−0.406 **	−0.416 **	−0.191 *

Abbreviations: TSK-TMD-S, Tampa Scale of Kinesiophobia-Temporomandibular Disorders; CF-PDI, Craniofacial Pain and Disability Index; TSK-11, Tampa Scale of Kinesiophobia-11 items (general kinesiophobia). * *p* < 0.05, ** *p* < 0.001.

**Table 5:** Diagnostic accuracy results and all optimal cut-off points.

Diagnostic Accuracy and Cut-Off Points	Subclinical	Mild	Moderate	Severe
Mean ± SD	16.88 ± 3.22	22.54 ± 1.76	26.75 ± 2.23	32.15 ± 2.99
95% CI	16.04 to 17.72	21.76 to 23.32	25.55 to 27.94	30.34 to 33.96
Cases, *n* (%)	59 (53.64%)	22 (20%)	16 (14.55%)	13 (11.82%)
Optimal cut-off	<23	≥23	≥26	≥31
Sensitivity (95% CI)	-	0.72 (0.49 to 0.89)	0.81 (0.54 to 0.96)	0.69 (0.38 to 0.91)
Specificity (95% CI)	-	1 (0.93 to 1)	1 (0.84 to 1)	1 (0.79 to 1)
Positive predictive value (95% CI)	-	1 (0.81 to 1)	1 (0.76 to 1)	1 (0.68 to 1)
Negative predictive value (95% CI)	-	0.91 (0.78 to 1)	0.88 (0.66 to 1)	0.80 (0.52 to 1)
